# When Resources Substitute for Each Other: How Emotional Intelligence and Organizational Support Interact in Relation to Resilience and Well-Being Among Healthcare Professionals

**DOI:** 10.3390/ejihpe15120254

**Published:** 2025-12-11

**Authors:** Wassim J. Aloulou

**Affiliations:** College of Business, Imam Mohammad Ibn Saud Islamic University (IMSIU), Riyadh 11432, Saudi Arabia; wjaloulou@imamu.edu.sa

**Keywords:** emotional intelligence, perceived organizational support, resilience, well-being, resource substitution, healthcare professionals, JD-R theory, COR theory, moderated mediation

## Abstract

The interaction between two critical resources, emotional intelligence (EI) and perceived organizational support (POS), is studied to understand how they come together to associate resilience and well-being among healthcare professionals. Based on the Conservation of Resources (COR) theory and the Job Demands–Resources (JD-R) model, we explore whether these resources are synergistic or whether there is a substitutive relationship when combined. Data were collected from 304 healthcare professionals in Saudi Arabia, both local and foreign. Using structural equation modeling, we examined a moderated mediation model in which resilience was examined as a mediator of the associations of EI and POS with well-being, and their interaction was included as a correlate of both resilience and well-being. EI and POS both individually showed positive association with resilience and well-being. However, in interaction, they significantly negatively associated with both resilience and well-being, suggesting a substitution effect—i.e., high levels of one resource are linked to a lower marginal value of the other. This suggests a nonlinear dynamic to resource accumulation among pressured healthcare workers. This study advances COR and JD-R theories by uncovering a substitution effect between emotional intelligence and organizational support, offering fresh insights into resource dynamics among healthcare professionals.

## 1. Introduction

Healthcare systems worldwide are undergoing increasing pressures, particularly in the aftermath of the COVID-19 pandemic, and the Kingdom of Saudi Arabia is no exception. In recent years, Saudi Arabia’s healthcare sector has experienced rapid transformation, driven by population growth, rising demand for specialized services, and the ambitious and bold reforms of Vision 2030. These reforms attempt to raise efficiency, improve patient outcomes, and boost both the public and private healthcare providers’ integration, digital health, and workforce development (Rahman & Al-Borie, 2021). At the same time, the sector relies heavily on a diverse workforce of Saudi nationals alongside a large proportion of expatriates, leading to unique working patterns in professional roles, cultural expectations, and organizational welfare structures (Elsheikh et al., 2018; Alonazi, 2020).

Healthcare professionals in Saudi Arabia have high job demands, such as long hours, heavy workloads, and emotional strain from intensive patient interactions. The COVID-19 pandemic further exacerbated these challenges, with evidence of elevated burnout, compassion fatigue, and decreased quality of life among the workforce (Alfaifi et al., 2024; Alreshidi & Rayani, 2023). These stressors not only weaken employee well-being but also endanger patient safety and healthcare delivery quality.

As the sector continues to expand, it has become a strategic priority to sustain the psychological resilience and well-being of healthcare professionals to safeguard personal well-being as well as promote organizational performance and patient care quality. Sustaining the resilience of healthcare staff has therefore become an emergent policy (Alonazi, 2020).

Given the growing importance of the sector and these pressing necessities, Saudi-based scholarship has predominantly focused on clinical performance, workforce localization, and institutional changes (Al-Asfour & Khan, 2014; Rahman & Al-Borie, 2021), not on psychosocial resources that enable healthcare professionals’ flourishing in this unique cultural and institutional context. The current literature suggests that emotional intelligence (EI), the ability to know, manage, and control emotions, is a personal resource that helps employees manage stress and adapt effectively (Schutte et al., 2007). Perceived organizational support (POS), feeling supported and valued by the organization, functions as a key contextual resource known to protect well-being and buffer strain (Rhoades & Eisenberger, 2002; Kurtessis et al., 2017). Both resources protect job strain and enhance resilience (Caesens & Stinglhamber, 2020; Alonazi, 2020) consistent with the logic of the Conservation of Resources (COR), which emphasizes the role of personal and contextual resources in sustaining well-being (Hobfoll et al., 2018).

Increasingly, scholars argue that EI supports employees’ ability to bounce back from adversity, whereas POS provides contextual protection; in highly supportive environments, the incremental benefit of EI and resilience may even become substitutive rather than purely additive (Bakker & Demerouti, 2017; Hobfoll et al., 2018). Despite this, EI and POS have been researched largely in isolation, positing additive influences. A key gap remains in understanding how personal and organizational resources are likely critical in sustaining resilience and interact in the unique Saudi healthcare context, where cultural diversity, high job demands, and rapid system reforms intersect.

Nonetheless, studies have seldom investigated how these resources interact with each other—whether their influences are additive, complementary, or even substitutive in increasing resilience and well-being, or whether, at times, they substitute each other, resulting in diminishing returns when both occur together. By not taking such interactions into account, current work might overestimate the straightforward resource dynamics that underlie resilience and well-being among Saudi Arabian healthcare professionals.

To bridge this gap is important not only for the construction of theoretical frameworks such as the Conservation of Resources (COR) theory and the Job Demands–Resources (JD-R) model but also for the development of evidence-informed interventions that suit the Saudi healthcare professionals’ context.

The paper is organized into key sections. The literature review develops the theoretical framework and its hypotheses. The methodology explains research design, sampling, measures, and analysis using structural equation modeling. The results report measurement validation and hypothesis testing. The discussion interprets findings and highlights theoretical and managerial implications. Finally, the limitations and future research section outlines constraints and directions for future research, and the conclusion summarizes the main insights of the study.

## 2. Literature Review

### 2.1. Theoretical Background

The Conservation of Resources (COR) theory and the Job Demands–Resources (JD-R) model provide robust theoretical foundations with which to explore how individual and organizational resources in combination impact resilience and well-being. COR theory proposes that individuals are motivated to acquire, maintain, and enhance resources, and stress arises when resources are lost, removed, or inadequately restored (Hobfoll, 1989; Hobfoll et al., 2018; Halbesleben et al., 2014). Emotional intelligence (EI) and resilience are in this sense critical personal resources, while perceived organizational support (POS) is a critical external resource. This framework is useful for explaining why EI and POS matter for resilience and well-being: both serve as resources within COR’s resource caravans (Halbesleben et al., 2014; S. Chen et al., 2015). Their interaction is theoretically defensible under COR theory.

JD-R theory complements COR by conceptualizing resources as motivational drivers that serve as buffers to demands at work, ultimately leading to well-being (Bakker & Demerouti, 2017). Interestingly, both models acknowledge that resource interactions are multidimensional with COR theory pointing to substitution or loss spirals and the JD-R model pointing to cross-level effects between job and personal resources (Y. L. Chen & Hsieh, 2023; Hu et al., 2011). This therefore enables us to consider not only the direct and mediating influence of resilience but also the moderating role of POS, providing an explanation that is sensitive to how resources can both add value and, perversely, detract from well-being when mixed. From this moderated mediation perspective, the paper offers specific and actionable recommendations for scholars and practitioners.

### 2.2. Hypothesis Development and Research Model

The well-being of healthcare professionals has become a critical concern in organizational psychology and health services research. Employee well-being is increasingly thought to be a complex construct, involving psychological, emotional, and occupational dimensions that directly influence work performance, patient safety, and organizational sustainability (Danna & Griffin, 1999; Page & Vella-Brodrick, 2009). In this literature, emotional intelligence (EI) and resilience are frequently identified as primary personal resources, while perceived organizational support (POS) represents a central organizational resource. The Conservation of Resources (COR) theory (Hobfoll, 1989; Hobfoll et al., 2018) and the Job Demands–Resources (JD-R) model (Bakker & Demerouti, 2017; Xanthopoulou et al., 2007) are called upon to account for how these resources relate to one another so they can be associated well-being among healthcare professionals.

Resilience or the ability to recover and adapt in adverse situations, has consistently been related to higher well-being across occupational contexts and organizational settings (Aloulou, 2022; Connor & Davidson, 2003; Hart et al., 2014). In healthcare, resilience reduces stress impacts and protects against burnout, fostering sustainable well-being despite high demands at work (Mealer et al., 2012; Brennan, 2017). Theoretically, resilience enables employees to gain access to coping and sustain psychological resources, enhancing satisfaction and reducing negative effects (Cooper et al., 2020). Empirically, the relationship between resilience and well-being was proven to be significant and positive (Lacomba-Trejo et al., 2022; Vilca-Pareja et al., 2022). Thus, it is hypothesized that resilience will be positively related to well-being (H1).

**H1.**  *Individual resilience is positively related to employee well-being.*

EI represents another significant personal resource, which is defined as the capacity for perceiving, regulating, and using emotions in oneself and others effectively (Mayer & Salovey, 1997). Extensive evidence from meta-analyses, systematic reviews, and empirical studies demonstrates that EI has been associated with different dimensions of well-being of university students, employees or managers: reduced stress, greater job satisfaction, higher psychological and subjective well-being, mental health, and satisfaction with life (Akanni et al., 2020; Di Fabio & Kenny, 2016; Extremera & Fernández-Berrocal, 2006; Extremera et al., 2020; Kotsou et al., 2019; Schutte et al., 2007; Carmeli et al., 2009; Higgs & Dulewicz, 2014). In the health sector, EI is particularly relevant since employees must manage their own emotions as well as discern their patients’ emotions often under pressure (Hashmi et al., 2024; Karimi et al., 2021). Thus, EI is expected to directly and positively predict well-being (H2).

**H2.**  *Emotional intelligence positively predicts employee well-being.*

Aside from its direct influences, EI is also expected to affect resilience. With increased EI, employees can better regulate emotions, positively reframe adversity, and use adaptive coping mechanisms and thereby strengthen resilience (Armstrong et al., 2011; Collado-Soler et al., 2023; Gameiro & Ferreira, 2024; Gey et al., 2025; Kawashima et al., 2025; Schneider et al., 2013; Trigueros et al., 2020; Valdez et al., 2023; Xing et al., 2023). Healthcare literature supports this relationship since it shows that emotionally intelligent professionals are resilient in high-stress environments (Delgado et al., 2017; Hashmi et al., 2024). EI is therefore hypothesized to have a positive prediction of resilience (H3).

**H3.**  *Emotional intelligence positively predicts individual resilience.*

Building on this, IRESI is also proposed to mediate EI and EWB. Previous studies show that EI enhances coping and recovery mechanisms, which also lead to greater well-being (Kong et al., 2019; Sánchez-Álvarez et al., 2016; Dobles Villegas & Schoeps, 2025; Eldeleklioglu & Yildiz, 2020; Kamboj & Garg, 2021; Kartol et al., 2024; Kenely, 2019; García-Martínez et al., 2022; Ngui & Lay, 2019; Pham et al., 2024). In other words, emotionally intelligent employees achieve greater well-being not only through direct emotional regulation but also through the resilience that helps them withstand stress and enhance the quality of their lives and well-being (García-Izquierdo et al., 2025; Qin et al., 2023; Shuo et al., 2022). This underpins the mediation hypothesis (H4).

**H4.**  *Individual resilience mediates the relationship between emotional intelligence and employee well-being.*

At the organizational level, a key contextual factor is POS. It refers to the degree to which employees believe that their organization values their effort and cares about their well-being (Eisenberger & Stinglhamber, 2011; Eisenberger et al., 2020). POS is generally linked to higher engagement, lower stress, work–life balance, and stronger well-being outcomes (Aloulou et al., 2023; Kurtessis et al., 2017; Köse et al., 2021). However, recent research suggests that organizational resources sometimes are utilized as substitutes for personal resources rather than being an addition to them (ten Brummelhuis & Bakker, 2012; Ramaci et al., 2024; Gallagher & Vella-Brodrick, 2008). When POS is high, employees may utilize their EI or IRESI less to maintain well-being, potentially weakening these relationships. Thus, POS is hypothesized to moderate the EI–IRESI relationship (H5) and the IRESI-EWB relationship (H6) in such a way that the positive associations weaken at higher levels of POS.

**H5.**  *Perceived organizational support moderates the relationship between emotional intelligence and resilience, such that the positive relationship is weaker when POS is high.*

**H6.**  *Perceived organizational support moderates the relationship between resilience and well-being, such that the positive relationship is weaker when POS is high.*

Specifically, the mediating role of IRESI should be lower in the case of high POS since it would be consistent with a resource substitution effect (Pierce & Aguinis, 2013; Mehta & Sharma, 2021; Maan et al., 2020). Finally, putting all the arguments together, POS is expected to moderate the indirect influence from EI to EWB through IRESI. In combination, COR and JD-R provide a nuanced framework to examine not only the direct and mediated resource influences but also resource interactions contingencies. This is as opposed to the presumption that “more is always better,” and positions us closer to being able to determine when and how resources jointly affect IRESI and EWB.

From the above, we can state the following hypotheses:

**H7.**  *The indirect influence of emotional intelligence on employee well-being via resilience is moderated by perceived organizational support.*

### 2.3. Research Model

**Figure 1:**
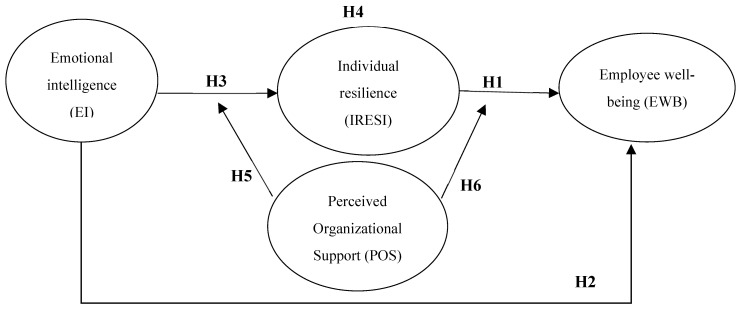
Hypothesized moderated mediation model of resources, resilience, and well-being. Source: Author’ work.

Figure 1 illustrates the hypothesized model, where EI is directly associated with IRESI and EWB, and IRESI mediates the EI–EWB relationship. POS moderates the EI–IRESI and IRESI–EWB paths, relating to conditional indirect effects.

## 3. Methods

### 3.1. Sampling and Data Collection

Data for this study were collected over a period of four months, from February to May 2025, using a structured survey administered to healthcare professionals in Saudi Arabia. An online questionnaire was developed through Google Forms and distributed using social media platforms and channels, including WhatsApp groups and LinkedIn profiles. This strategy allowed access to different segments of healthcare professionals, such as physicians, nurses, technicians, and other allied professionals, with coverage attained across a variety of roles within the healthcare sector. As there is no national database of healthcare professionals available, non-probability sampling techniques were applied. Convenience sampling was applied in combination with snowball sampling, where the first respondents were allowed to share the link of the survey with colleagues in their professional network.

Following the data collection period, rigorous data screening procedures were conducted. In line with Tabachnick and Fidell’s (2013) recommendations, the dataset was screened for missing data and potential outliers, none of which were detected. This ensured the integrity and reliability of the final sample. After data cleansing, 304 valid responses were retained for analysis, which provided an adequate sample size for structural equation modeling and hypothesis testing. The sample size exceeded the recommended thresholds for SEM (minimum sample size of 200, e.g., Collier, 2020), which also added to the validity of the study’s statistical evaluation.

Table 1 showing the demographic profile gives a general picture of the structure of the sample. The respondents are predominantly young, with over 70% aged under 40 years, and early in their careers as would be clear from concentrated work experience of less than five years. The workforce is predominantly male (69%) and unmarried, with minimal family responsibilities. Bachelor’s degree holders are the biggest group educationally, while higher and lower qualifications also feature.

Full-time work prevails, and shift work is prevalent, showing the demanding nature of the industry. Two-thirds of the sample are Saudis, with expatriates bringing considerable diversity. In terms of occupation, physicians, nurses, technicians, and other professionals are quite evenly spread.

Table 2 of organizational types reflects a public sector dominance (55%), though private institutions also account for nearly one-third of the sample. Most organizations operate in Riyadh (66%) due to the city’s central location in healthcare delivery. Hospital organizations form the largest organizational category (49%), followed by medical complexes (18%) and primary care centers (12%), providing varied institutional settings. Smaller segments include laboratories, dental centers, and other sites, contributing to a diversified organizational composition. This distribution reflects a diversified sample that includes both private and public healthcare environments, and health care service types.

### 3.2. Instruments

All constructs in the study were measured on a 5-point Likert scale, ranging from 1 (“strongly disagree”) to 5 (“strongly agree”).

*Independent Variable—Emotional intelligence (EI)*: A 16-items scale developed by Wong & Law (2002) was used to measure EI. It comprises four dimensions: intrapersonal perception (self-emotion appraisal), interpersonal perception (others’ emotion appraisal), assimilation (use of emotions), and emotional regulation. For each dimension, a sample of item includes: “I have good understanding of my own emotions”, “I always know my friends’ emotions from their behavior”, “I always tell myself I am a competent person”, “I am quite capable of controlling my own emotions”. This scale was validated in the context of Portugal and Saudi Arabia (Gameiro & Ferreira, 2024; Alsughayir, 2021).

*Moderator Variable—Perceived organizational support (POS)*: This variable was measured using the scale of ten items developed by Eisenberger et al. (2020). A sample item includes: “The organization strongly considers my goals and values”. This scale was also validated in the Saudi Context with Masalat et al. (2025) and Siraj et al. (2023).

*Mediator—Individual resilience (IRESI):* This variable was measured using the Connor–Davidson resilience scale 10 (CD-RISC 10) developed by Connor and Davidson (2003) and validated by Campbell-Sills and Stein (2007), Levey et al. (2021) and Siraj et al. (2023). A sample item includes “Coping with stress can strengthen me”.

*Dependent Variable—Employee well-being (EWB):* This variable was measured by an 18-item scale developed by Zheng et al. (2015). Employee well-being consisted of three dimensions: life well-being with 6 items (e.g., “I feel satisfied with my life”), work well-being with 6 items (e.g., “I am satisfied with my work responsibilities.”), and psychological well-being with 6 items (e.g., “I feel I have grown as a person”). One dimension or more were tested and validated in previous studies (e.g., Aloulou et al., 2025; Langove et al., 2023).

Since the adapted measurement scales from the literature were originally developed and validated in English, including in several studies using Saudi samples, the survey was conducted in English without translation. Furthermore, English is the main professional and educational language in Saudi healthcare settings, which makes the items consistently reliable and limits the potential effects on validity of the original measures.

### 3.3. Examination of the Reliability and Validity of the Study’s Variables

The results presented in Table 3 reveal good validity and reliability of variables. The factor loadings are far above the minimum acceptable figure of 0.60, confirming good indicator reliability. All KMO values are greater than 0.90, signifying adequacy of sample and suitability for factor analysis. Internal consistency is confirmed using Cronbach’s alpha ranging from 0.935 to 0.952, far better than the suggested 0.70. Composite reliability (CR) statistics are also 0.945 and 0.959, thus determining the measurement scales’ reliability. Moreover, average variance extracted (AVE) statistics are between 0.568 and 0.700, which is above the 0.50 threshold and thus determining convergent validity. Overall, all the above determinations ascertain that the measurement model is both valid and reliable and thus a good foundation on which to perform subsequent structural equation modeling and hypothesis testing.

The Fornell–Larcker results confirm discriminant validity among the study constructs in Table 4 by comparing the square root of the AVE for each construct (diagonal values) with the corresponding inter-construct correlations. For example, EI’s AVE square root (0.754) is higher than its correlations with IRESI (0.678), POS (0.552), and EWB (0.637). Similar patterns hold for other constructs, showing clear distinctiveness among EI, IRESI, POS, and EWB.

### 3.4. Common Method Bias Analysis

Common method bias was tested using both Harman’s single factor test and confirmatory factor analysis (CFA) (MacKenzie & Podsakoff, 2012). In the unrotated factor solution, the first factor accounted for a mere 43.03% of variance, which is below the significant 50% critical point, and seven factors combined accounted for 65.46%. In addition, CFA results indicated (Table 5) that the poor fit of the one-factor model (CMIN/DF = 4.299, CFI = 0.653, RMSEA = 0.104), whereas hypothesized expected four-factor model was a good fit (CMIN/DF = 1.689, CFI = 0.930, RMSEA = 0.048). These collectively determine that common method bias is not a significant problem in this research (Hair et al., 2019).

## 4. Results

Once measurement model was established to have good fit, collinearity diagnostics were tested for the moderated mediation model. The values indicated a VIF between 1.000 and 2.283 (al l < 5), condition index of 2.809 (<30), and a Durbin–Watson statistic of 2.064, so multicollinearity did not pose a problem. Structural equation modeling (SEM) with AMOS (Version 21.0) was then applied to test the theory framework presented in Figure 1. Three steps were involved in the analysis: one was to test the direct association between the core constructs, and the second was to test mediation and moderation to represent indirect relationships. Path analysis results are reported in Table 6.

This table outlines unstandardized and standardized coefficients (β), standard errors (S.E.), critical ratios (C.R.), and *p*-values of every hypothesized relationship. Taken collectively, these results clarify the strength and significance of the relationships within the conceptual model and signify which hypotheses were empirically supported. It summarizes the findings of a moderated-mediation model exploring how EI and POS affect IRESI and EWB, including their interactive effects.

### 4.1. Direct Relationships

Hypotheses of direct relationships were supported. In fact, POS → IRESI (β = 0.267, *p* < 0.001) and EI → IRESI (β = 0.667, *p* < 0.001) both show strong, positive influences, confirming that personal and organizational resources independently enhance employees’ capacity to bounce back from stress. In addition, POS → EWB (β = 0.207, *p* < 0.001) and EI → EWB (β = 0.147, *p* = 0.015) also positively and significantly predict EWB, aligning with the JD-R and COR models which suggest that personal and organizational resources facilitate positive psychological outcomes. For instance, IRESI → EWB (β = 0.348, *p* < 0.001) is a particularly strong path, indicating that IRESI acts as a central mechanism through which both EI and POS contribute to overall EWB.

### 4.2. Mediation Analysis

A mediation analysis was carried out following the procedures described by Collier (2020), which necessitate examining the associations between all variables in the mediation process. For the mediation analysis, bootstrapping was performed to test the indirect effects of EI on EWB. The number of bootstrap samples is 2000 with 95 bias-corrected confidence intervals.

This mediation analysis examines whether IRESI mediates the relationship between EI and EWB. Table 7 shows the results of this mediation analysis: the direct, indirect and total effects of the independent variable on dependent variable through the mediator.

EI has a significant direct influence on EWB (Direct effect of EI on EWB: 0.159, *p* < 0.05), suggesting that emotionally intelligent healthcare professionals are better equipped to manage emotional challenges and sustain well-being. The mediation path through IRESI is statistically significant and stronger than the direct path (Indirect effect via resilience: 0.251, *p* < 0.05), indicating that EI substantially contributes to EWB by enhancing IRESI. The total effect confirms that EI plays a meaningful and positive role in promoting EWB, both directly and indirectly (Total effect: 0.410, *p* < 0.05).

The persistence of a significant direct effect alongside a significant indirect effect suggests that IRESI partially explains the relationship between EI and EWB. EI not only builds IRESI but also independently supports EWB through its specific mechanisms (e.g., emotion regulation).

### 4.3. Moderation Analysis

The interaction term EI × POS → IRESI (β = −0.125, *p* < 0.001) reveals a significant negative interaction, suggesting a resource substitution effect. In other words, when both EI and POS are high, their combined influence on IRESI is weaker than expected, potentially due to redundancy or interference. Similarly, IRESI × POS → EWB (β = −0.080, *p* = 0.006) also shows a negative interaction, indicating that the benefit of IRESI on EWB diminishes at higher levels of POS.

**Figure 2:**
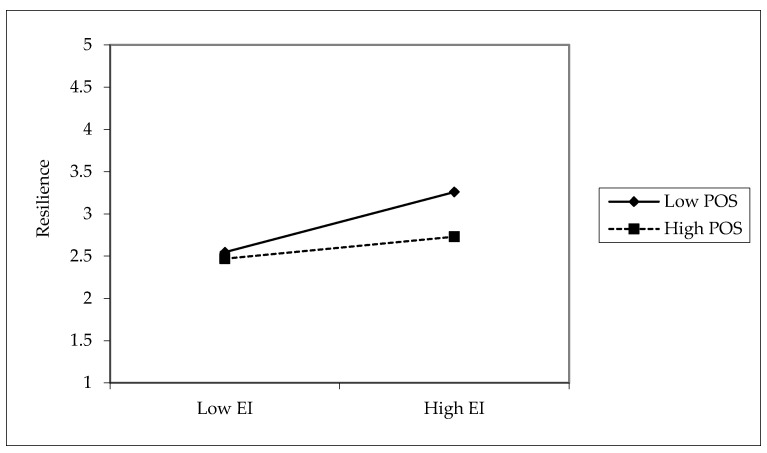
Interaction plot EI × POS—IRESI relationship.

Figure 2 shows that EI more strongly predicts IRESI under low-POS conditions, while this effect weakens when POS is high. This suggests a substitution effect, where POS reduces the marginal benefit of EI in building IRESI. In other words, employees rely more on their personal resources (EI) when POS is lacking, but when support is abundant, EI becomes less critical in building IRESI. This provides evidence against a purely additive effect of resources and supports the “too much of a good thing” perspective in the resource literature.

**Figure 3:**
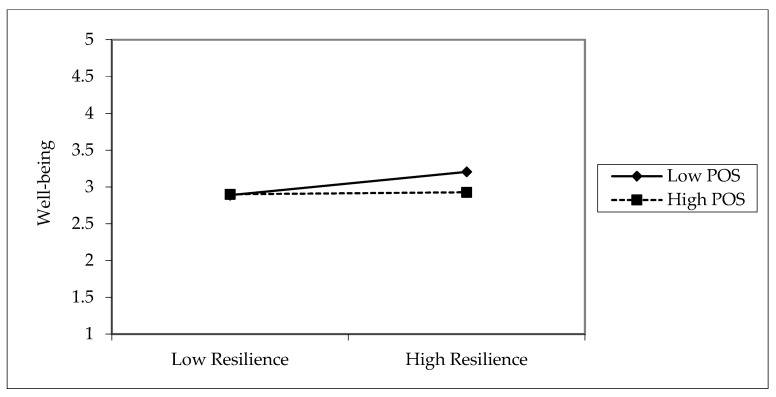
Interaction plot IRESI × POS—EWB relationship.

Figure 3 shows that this interaction plot reveals how the effect of IRESI on EWB differs across low and high levels of POS. in fact, IRESI improves EWB more under low-POS conditions than under high POS, where the effect is negligible (Slope is flat). This indicates that POS substitutes the role of IRESI: when support is lacking, IRESI is crucial for maintaining EWB, but when support is strong, its added value is minimal.

### 4.4. Moderated Mediation Analysis with PROCESS

To examine the moderated mediation model using PROCESS Model 58 (Hayes, 2022), EI was specified as the independent variable, IRESI as the mediator, and EWB as the dependent variable. POS was included as a moderator on both the a-path (EI → IRESI) and the b-path (IRESI → EWB). Variables were mean-centered (Var_c) to facilitate interpretation of interactions, and bootstrapping with 5000 resamples was applied to obtain bias-corrected confidence intervals for indirect effects. PROCESS estimated the direct influence of EI on EWB, the conditional effects of EI on IRESI, and IRESI on EWB at different levels of POS, as well as the conditional indirect effect through IRESI. Interaction terms were examined for significance, and simple slopes were plotted to interpret the moderation. This approach allows for assessing whether the mediating role of IRESI varies depending on POS, providing a comprehensive understanding of the interplay between EI, IRESI, POS, and IWB.

PROCESS Model 58 (Hayes, 2022) revealed a significant moderated mediation pattern. EI positively predicted IRESI, and this association was significantly moderated by POS (β = −0.108, *p* = 0.001). Conditional effects indicated that EI had the strongest effect on IRESI under low POS (β = 0.456), a moderate effect under mean POS (β = 0.363) and the weakest effect under high POS (β = 0.270), suggesting that POS substitutes for EI as a resource. Similarly, the IRESI–EWB link was moderated by POS (β = −0.091, *p* = 0.005), with IRESI exerting a stronger effect on EWB when POS was low. Importantly, the indirect effect of EI on EWB through IRESI varied across levels of POS, confirming a significant moderated mediation. Accordingly, hypothesis H7 is also confirmed.

The indirect effect was strongest at low POS (effect = 0.285), weaker at mean POS (0.187), and weakest at high POS (0.110), with all confidence intervals excluding zero. Overall, these findings indicate that POS dampens both stages of mediation, meaning that the contribution of EI and IRESI to EWB becomes less influential when employees already perceive strong organizational support. Table 8 summarizes the results of the conditional indirect effects of EI on EWB.

## 5. Discussion and Implications

### 5.1. Discussion

This study examined the influence of EI on EWB through IRESI, with POS as a boundary condition. The findings confirmed the direct associations among EI, IRESI and EWB documented in previous studies (Schutte et al., 2007; Brennan, 2017; Gameiro & Ferreira, 2024). The findings also confirmed that EI predicts IRESI positively, which in turn boosts EWB. This indirect effect emphasizes the significance of IRESI as a psychological mechanism reframing emotional capabilities into healthier outcomes. Earlier studies support this stance: emotionally intelligent individuals can more easily recognize and regulate their own emotions, which facilitates adaptive coping and resilience (Cazan & Năstasă, 2015; Kotsou et al., 2019). IRESI has, in turn, been consistently linked to greater psychological well-being and lower burnout (Hart et al., 2014).

Most importantly, POS moderated the relationship between IRESI and EWB, with greater effects observed at low POS levels. This suggests a resource-substitution mechanism consistent with the Conservation of Resources Theory (Hobfoll et al., 2018; Shantz et al., 2016), wherein personal resources (e.g., IRESI) compensate for the lack of contextual support in organizational settings. As a contrast, if POS is high, employees may rely less on IRESI, as organizational support provides the safety net to guarantee well-being. This finding challenges the additive-resource assumption, which has otherwise positioned POS as always being positive together with individual traits and their aggregation should consistently enhance resilience and well-being (Rhoades & Eisenberger, 2002; Schaufeli & Taris, 2014). Our results go against the traditional view and align more closely with interactionist perspectives that suggest resources can substitute for one another in context-dependent manners (Halbesleben et al., 2014; ten Brummelhuis & Bakker, 2012).

The results add to the development of arguments about whether EI exerts a direct effect on EWB or acts primarily indirectly through mediators. While some scholars argue for a direct pathway (Kotsou et al., 2019), our findings align with those highlighting indirect effects through individual resilience (Keefer et al., 2018). IRESI is thus an essential explanatory link, especially for emotionally challenging and demanding sectors such as healthcare.

Together, these findings display complex picture: EI builds IRESI, IRESI adds to EWB, and POS affects the strength of this pathway. Such interaction encourages insight into how personal and organizational resources together affect employees’ psychological outcomes. Specifically, the conditional indirect effects suggest that the association between EI and EWB through IRESI varies across levels of POS, with higher POS linked to a weaker incremental association of EI—reflecting a potential substitution effect between personal and organizational resources.

### 5.2. Theoretical Implications

This study has several theoretical contributions. First, it reinforces Conservation of Resources (COR) theory (Hobfoll, 1989), positioning EI as a personal resource through which individuals build resilience and defend well-being. By establishing IRESI as a mediator, it refines models that traditionally treat EI as a direct predictor. Second, POS moderation offers proof for the resource substitution hypothesis: IRESI exerts a larger influence when POS is scarce but weaker when there is abundant support. This contributes to existing literature that tends to place POS as consistently beneficial (Rhoades & Eisenberger, 2002), showing instead that its value depends on the availability of other resources (Maan et al., 2020). Third, the study challenges the Job Demands–Resources (JD-R) model’s assumption of additive effects and instead suggests that conjoint resources can create redundancy or declines in returns. This perspective aligns with ten Brummelhuis and Bakker’s (2012) call for dynamic resource interaction models and Too-Much-of-Good-Thing research (Pierce & Aguinis, 2013), cautioning against assuming “more is always better”.

Finally, the findings integrate EI, IRESI, and POS into a more nuanced resource framework, highlighting context-dependent and conditional effects. This offers a new contribution through illustration that resources interact in complex ways that influence healthcare workers’ well-being, moving theoretical debates ahead of linear models.

### 5.3. Managerial Implications

The results have several implications for healthcare organizations. First, as EI is a source of IRESI, managers need to prioritize EI development programs like emotional regulation workshops, peer coaching, and web-based training modules. The evidence confirms that EI can be developed over time, with spillovers that are positive on coping and well-being (Hodzic et al., 2017).

Second, resilience needs to be cultivated deliberately through formal (micro)interventions, including stress management training, mindfulness practices, and peer support at team level. Such approaches have been suggested to buffer emotional exhaustion and support mental health outcomes according to existing evidence (West et al., 2016; Benavides-Gil et al., 2024; Šimunjak, 2023). Web-based resilience interventions are also found to be acceptable for healthcare contexts, though between trials there is inconsistency of effect (Henshall et al., 2023).

Third, POS moderation suggests support needs to be calibrated rather than maximized. Excessive POS may inadvertently blunt the motivational function of personal resilience, while insufficient POS forces employees to rely solely on their inner resources. Managers would thus need to tailor support through structural policies (e.g., flexible scheduling, participatory decision-making) as well as empowerment strategies that encourage employees to leverage and develop their emotional and resilience capacities (Eisenberger & Stinglhamber, 2011).

Finally, HR practices need to consider employee profiles: those lower in resilience would require more solid organizational support, while highly resilient individuals may thrive with moderate support and more autonomy. Differentiated interventions will ensure that resources are not redundant but complement each other, optimizing both well-being and organizational performance.

## 6. Limitations and Future Research Perspectives

Notwithstanding its contributions, this study has several limitations that need to be mentioned. The first is that the cross-sectional design limits causal inferences between EI, IRESI, POS, and EWB. Although structural equation modeling provides evidence of directionality, longitudinal designs will need to establish how these relationships evolve over time, especially given the dynamic nature of well-being within high-stress contexts like healthcare. Second, the reliance on self-reported measures may cause common method bias, even though statistical checks suggest that it is not a severe problem. Future research would be strengthened by the inclusion of multisource data, such as supervisor ratings of EI or objective well-being indicators, to preclude subjectivity. Third, convenience and snowball samplings may reduce generalizability because the sample can never stand for all healthcare professionals in Saudi Arabia, particularly those in remote regions or non-hospital settings or older professionals less likely to respond to online surveys. Expanded sampling frames (e.g., institutional) and the use of probability-based methods would enhance representativeness. Last, this study focused on a single cultural context; cross-cultural comparisons could illuminate whether the observed substitution effect of POS and IRESI is culturally specific or universal. Finally, only EI, POS, IRESI, and EWB were entered into the model. Future research might include other variables, such as job demands, leadership styles, or digital health resources, to capture a wider view of resource dynamics in health care environments. Such additions would enable further insight into how organizational and personal factors jointly affect EWB.

## 7. Conclusions

This study provides profound understanding of the relationship between emotional intelligence, perceived organizational support, and resilience in influencing healthcare professionals’ well-being. By providing evidence of IRESI mediating EI and well-being, and POS moderating the relationship between IRESI and EWB, the findings suggest a nuanced resource substitution mechanism. IRESI has a greater role to play in preserving EWB under low POS, but under healthy settings, IRESI is less critical. These results enrich COR theory and challenge assumptions about the additive effects of personal and organizational resources. From a practical perspective, the findings identify the worth in developing both personal capacities, such as EI and resilience, and contextual support, such as organizational policies and managerial practices, to foster well-being among healthcare workers. While healthcare systems are facing mounting stresses, particularly in demanding environments like Saudi Arabia, organizations that strategically balance resource building and institutional support will be best positioned to protect and foster employee well-being.

## Figures and Tables

**Table 1:** Respondents’ demographics.

Respondent’s Characteristics		Frequency	Percent %
Age			
	Less than 22 years old	47	15.46
	Between 23 and 30 years old	126	41.45
	Between 31 and 40 years	87	28.62
	Between 41 and 50 years	35	11.51
	51 years and more	9	2.96
Gender			
	Male	209	68.75
	Female	95	31.25
Marital status			
	Single	185	60.86
	Married	110	36.18
	Divorced/Widowed	9	2.96
Number of Children in charge			
	0	190	62.50
	1–2	65	21.38
	3–4	31	10.20
	5 and more	18	5.92
Educational level			
	Diploma	34	11.18
	Bachelor	178	58.55
	Master	53	17.43
	Ph.D.	39	12.83
Work experience			
	−1 year	64	21.05
	Between 1 and 5 years	110	36.18
	Between 5 and 10 years	61	20.07
	Between 10 and 15 years	41	13.49
	+15 years	28	9.21
Employment status			
	Full-time	235	77.30
	Part-time	34	11.18
	Other	35	11.51
Shifting work			
	Fixed	124	40.79
	Rotating	180	59.21
Nationality			
	Saudis	201	66.12
	Expatriates	103	33.88
Job			
	Nurse	73	24.01
	Physician	91	29.93
	Technician	65	21.38
	Other allied healthcare worker	75	24.67

Source: Author’ work.

**Table 2:** Healthcare organizations’ demographics.

Healthcare Organization’s Characteristics		Frequency	Percent %
Sector			
	Public sector	168	55.26
	Private sector	99	32.57
	Other	37	12.17
Localization of the healthcare organization			
	In Riyadh	202	66.45
	Outside Riyadh	102	33.55
Healthcare organization			
	Primary health care center	36	11.84
	Hospital	148	48.68
	Medical Complex/Aggregate Clinics	55	18.09
	Medical laboratory	17	5.59
	Dental center	14	4.61
	Others	33	10.86

Source: Author’ work.

**Table 3:** Reliability and validity of the study’s variables.

Variables	Dimensions	Factor Loadings	%	KMO	Alpha	CR	AVE
Emotional intelligence (EI)	- Self-emotion appraisal (EI1–EI4) - Others’ emotion appraisal (EI5–EI8) - Use of emotion (EI9–EI12) - Regulation of emotion (EI13–EI16)	From 0.661 to 0.789	56.819	0.956	0.949	0.955	0.568
Individual resilience (IRESI)	IRESI1-IRESI10	From 0.765 to 0.845	63.377	0.957	0.935	0.945	0.634
Perceived Organizational Support (POS)	POS1-POS10	From 0.765 to 0.872	69.919	0.956	0.952	0.959	0.700
Employee Well-being (EWB)	- Life well-being (EWB1–EWB6) - Work Well-being (EWB9–EWB12) (*) - Psychological Well-being (EWB13–EWB18)	From 0.661 to 0.823	57.831	0.956	0.951	0.956	0.578

(*) EWB7 and EWB8 were dropped from the analysis for lower factor loadings. Source: Author’ work.

**Table 4:** Correlation matrix and discrimination validity with Fornell and Larcker (1981) criterion.

	EI	IRESI	POS	EWB
EI	0.754			
IRESI	0.678 **	0.796		
POS	0.552 **	0.572 **	0.837	
EWB	0.637 **	0.679 **	0.606 **	0.760

** *p* < 0.01. Source: Author’ work.

**Table 5:** One-Factor vs. Four-Factor Model Fit.

Model Fit Indices	CMIN/DF	RMR	GFI	IFI	TLI	CFI	RMSEA
One factor	4.299	0.120	0.431	0.654	0.639	0.653	0.104
Four factors	1.671	0.079	0.871	0.948	0.940	0.948	0.048

Source: Author’ work.

**Table 6:** Path analysis.

Hypothesized Relationship			U_Estimate	S_Estimate	S.E.	C.R.	*p*	Hypothesis
EWB	←	IRESI	0.348 ***	0.444	0.059	5.940	***	H1 supported
EWB	←	EI	0.147 *	0.159	0.061	2.425	0.015	H2 supported
IRESI	←	EI	0.667 ***	0.565	0.077	8.622	***	H3 supported
IRESI	←	POS	0.267 ***	0.290	0.051	5.281	***	-
EWB	←	POS	0.207 ***	0.286	0.042	4.984	***	-
IRESI	←	EI × POS	−0.125 ***	−0.136	0.037	−3.396	***	H5 supported
EWB	←	IRESI × POS	−0.080 **	−0.109	0.029	−2.766	0.006	H6 supported

CMIN/DF = 1.689; RMR = 0.066; GFI = 0.794; IFI = 0.926; TLI = 0.920; CFI = 0.926; RMSEA = 0.048. U_Estimate: unstandardised estimate; S_Estimate: standardised estimate. *** *p* < 0.001; ** *p* < 0.01; * *p* < 0.05. Source: Author’ work.

**Table 7:** Mediation analysis.

IV	Mediator	DV	Direct	Indirect	Total	Hypothesis H4
EI	IRESI	EWB	0.159 *	0.251 *	0.410 *	Partial mediation

* *p* < 0.05; Source: Author’ own work.

**Table 8:** Conditional indirect effects of EI on EWB.

Indirect Effect: EI_c → IRESI_c → EWB				
POSm_c	Effect	BootSE	BootLLCI	BootULCI
−1.0222	0.2854	0.0597	0.1735	0.4038
0.0000	0.1874	0.0403	0.1135	0.2707
1.0222	0.1097	0.0368	0.0443	0.1890

Extracted from the PROCESS procedure V4.2. Source: Author’s own work.

## Data Availability

Data are available upon request from the researcher.

## Abbreviations

The following abbreviations are used in this manuscript:

EI	Emotional Intelligence
POS	Perceived Organizational Support
IRESI	Individual Resilience
EWB	Employee Well-Being
